# Supplementary material for: Age-dependent loss of adipose Rubicon promotes metabolic disorders via excess autophagy
Source: Nat Commun. 2020 Aug 18;11:4150. doi: 10.1038/s41467-020-17985-w (PMC7434891; doi:10.1038/s41467-020-17985-w)
Supplement: Supplementary file 1 — Supplementary Information [file 41467_2020_17985_MOESM1_ESM.pdf]

**Yamamuro, T. *et al*. Age-dependent loss of adipose Rubicon promotes metabolic disorders via excess autophagy**

Supplementary Materials: 9 Figures; 1 Table

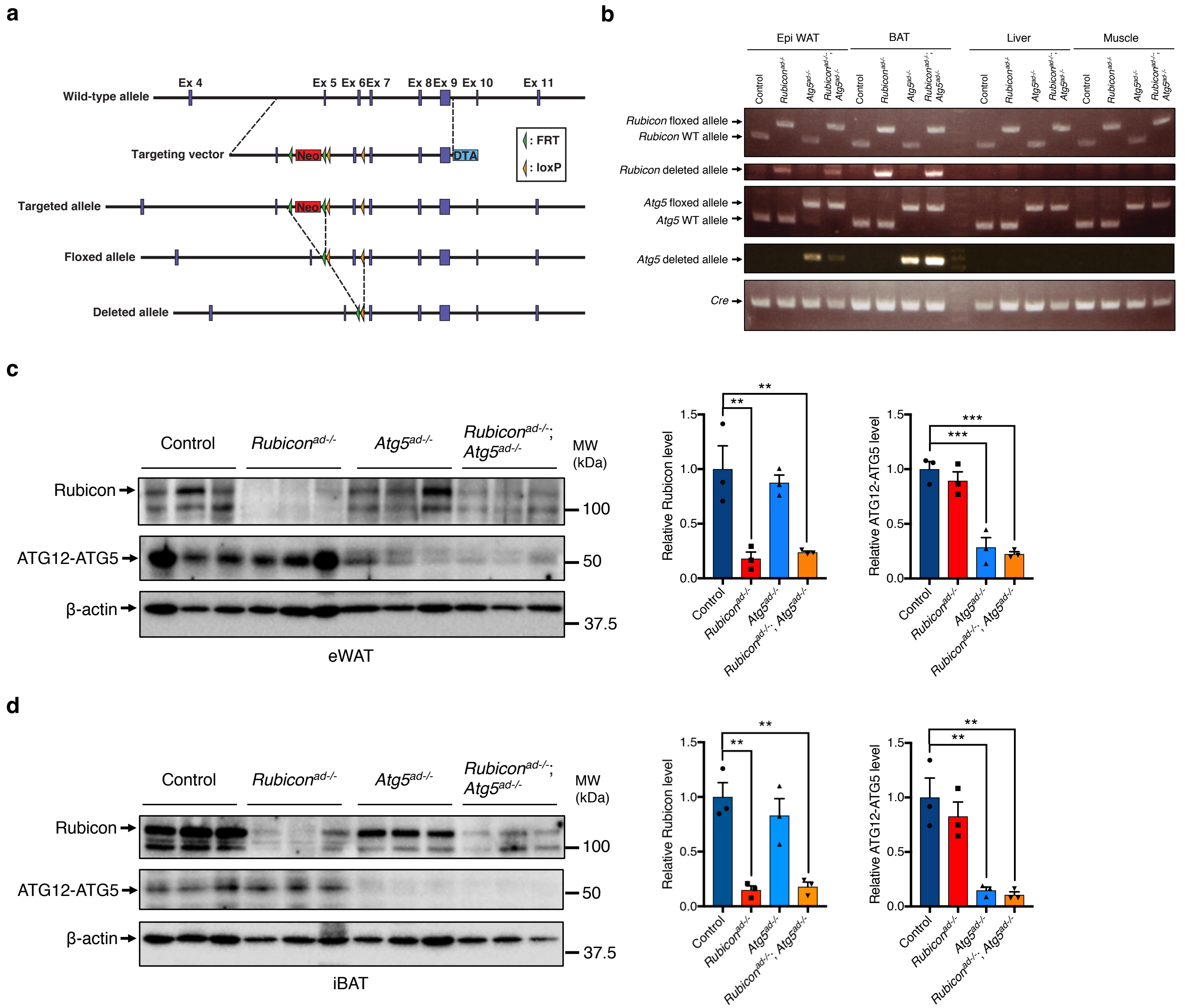
a
Ex 4
Ex 5
Ex 6
Ex 7
Ex 8
Ex 9
Ex 10
Ex 11
Wild-type allele
Targeting vector
Neo
DTA
: FRT
: loxP
Targeted allele
Floxed allele
Deleted allele
b
Epi WAT
BAT
Liver
Muscle
Control
Rubicon ad-/-
Atg5 ad-/-
Rubicon ad-/-; Atg5 ad-/-
Rubicon floxed allele
Rubicon WT allele
Rubicon deleted allele
Atg5 floxed allele
Atg5 WT allele
Atg5 deleted allele
Cre
c
Control
Rubicon ad-/-
Atg5 ad-/-
Rubicon ad-/-; Atg5 ad-/-
MW (kDa)
Rubicon
ATG12-ATG5
β-actin
100
50
37.5
eWAT
Relative Rubicon level
Relative ATG12-ATG5 level
1.5
1.0
0.5
0.0
**
***
d
iBAT

**Supplementary Figure 1** Generation of adipose-specific *Rubicon*-knockout mice. **a** Scheme describing the generation of *Rubicon* knockouts. **b** PCR Genotyping. Adipocyte-specific Cre-recombination is confirmed by PCR amplification of each locus in the eWAT, iBAT, liver, and muscle of 21-week-old mice of the indicated genotypes. Notably, we used *Adipoq*-*Cre* mice as controls to exclude the possibility that *Cre* recombinase affects basic cellular function[70]. n = 3 independent experiments with similar results. **c, d** Immunoblotting to detect Rubicon or ATG12–ATG5 complex in eWAT (**c**) or iBAT (**d**) depots of 21-week-old mice of the indicated genotypes. n = 3 mice. Quantification data is shown in the graphs at the right of each blot. Error bars indicate means ± SEM. Data were analysed by one-way ANOVA followed by Tukey's test (**c, d**). *P* value from top to bottom and left to right: 0.0073, 0.0047, 0.0002, 0.0004 (**c**), 0.0024, 0.0019, 0.0023, 0.0030 (**d**). *$P < 0.05$, **$P < 0.01$, ***$P < 0.001$, ****$P < 0.0001$. N.S., not significant.

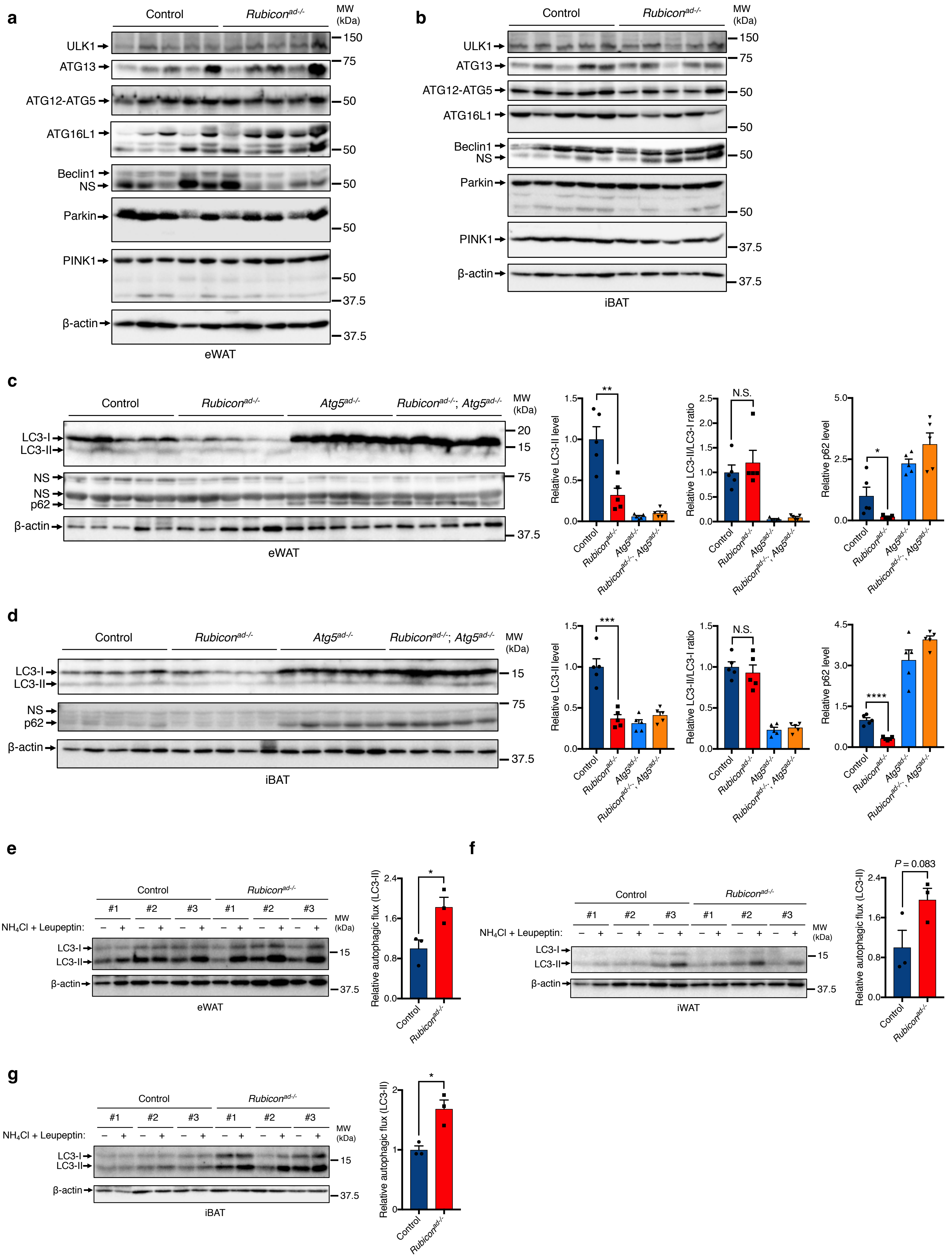
a
Control
Rubicon^ad-/-
MW (kDa)
ULK1
ATG13
ATG12-ATG5
ATG16L1
Beclin1
NS
Parkin
PINK1
β-actin
150
75
50
37.5
eWAT
b
iBAT
c
LC3-I
LC3-II
p62
Atg5^ad-/-
Rubicon^ad-/-; Atg5^ad-/-
20
15
Relative LC3-II level
Relative LC3-II/LC3-I ratio
Relative p62 level
N.S.
**
***
****
*
d
e
f
g
#1
#2
#3
NH4Cl + Leupeptin:
Relative autophagic flux (LC3-II)
P = 0.083
iWAT

**Supplementary Figure 2** Specific knockout of *Rubicon* in adipocytes leads to an increase in basal autophagy. **a, b** Immunoblotting to detect the indicated proteins in eWAT (**a**) or iBAT (**b**) depots of 21-week-old mice of the indicated genotypes on an NCD. n = 5 mice. **c, d** Immunoblotting to detect LC3-II and p62 in eWAT (**c**) or iBAT (**d**) depots of 21-week-old mice of the indicated genotypes on an NCD. n = 5 mice. **e–g** *Ex vivo* autophagic flux assay based on LC3-II degradation in eWAT (**e**), iWAT (**f**), and iBAT (**g**) depots of 24-week-old mice of the indicated genotypes on an NCD. n = 3 mice. Quantification data is shown in the graphs at the right of each blot. Error bars indicate means ± SEM. Data were analysed by two-tailed Student's *t*-test (**c–g**). *P* value from left to right: 0.0047, 0.5172, 0.0482 (**c**), 0.0004, 0.5546, < 0.0001 (**d**), 0.0332 (**e**), 0.0833 (**f**), 0.0137 (**g**). $^{*}P < 0.05$, $^{**}P < 0.01$, $^{***}P < 0.001$, $^{****}P < 0.0001$. N.S., not significant.

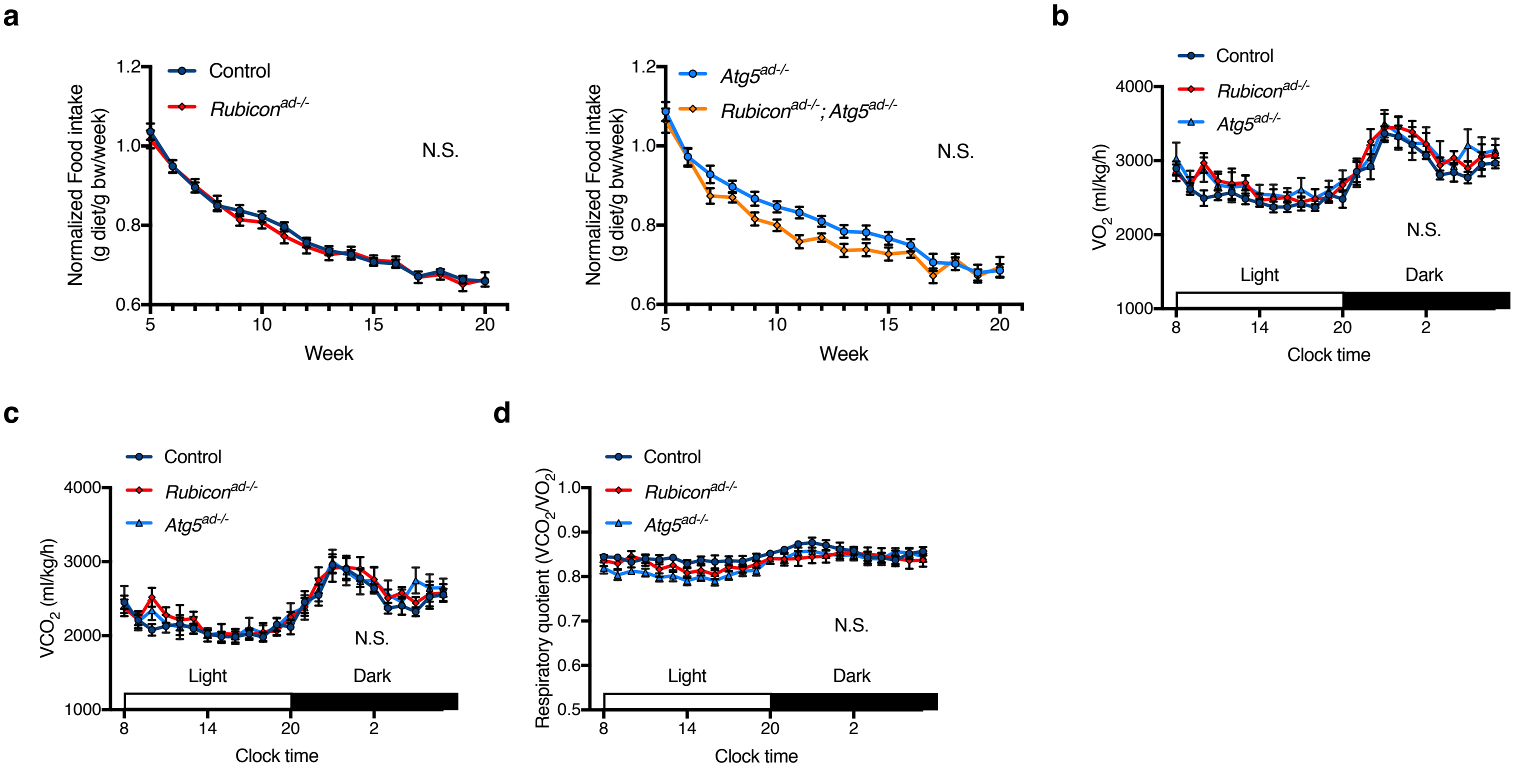
a
Normalized Food intake
(g diet/g bw/week)
Control
Rubicon^ad-/-
N.S.
Week
Atg5^ad-/-
Rubicon^ad-/-; Atg5^ad-/-
N.S.
Week
b
Control
Rubicon^ad-/-
Atg5^ad-/-
VO2 (ml/kg/h)
N.S.
Light
Dark
Clock time
c
Control
Rubicon^ad-/-
Atg5^ad-/-
VCO2 (ml/kg/h)
N.S.
Light
Dark
Clock time
d
Control
Rubicon^ad-/-
Atg5^ad-/-
Respiratory quotient (VCO2/VO2)
N.S.
Light
Dark
Clock time

**Supplementary Figure 3** Deletion of *Rubicon* or *Atg5* in adipocyte has no impact on food intake or oxygen consumption. **a** Normalised food intake of mice of the indicated genotypes, described in Fig. 1b. Food intake was monitored weekly. Control, n = 35; *Rubicon*$^{ad-/-}$, n = 21; *Atg5*$^{ad-/-}$, n = 27; *Rubicon*$^{ad-/-}$; *Atg5*$^{ad-/-}$, n = 20. **b–d** Whole-body $O_2$ consumption (**b**), $CO_2$ production (**c**), and RQ (**d**) of 23–24-week-old mice of the indicated genotypes on an NCD. Control, n = 8; *Rubicon*$^{ad-/-}$, n = 8; *Atg5*$^{ad-/-}$, n = 9. Error bars indicate means ± SEM. Data were analysed by two-way repeated-measures ANOVA followed by Fisher's LSD test (**a**) or two-way repeated-measures ANOVA followed by Tukey's test (**b–d**). *P* value from left to right: 0.7111, 0.1467 (**a**), 0.6089 (**b**), 0.8519 (**c**), 0.1947 (**d**). N.S., not significant.

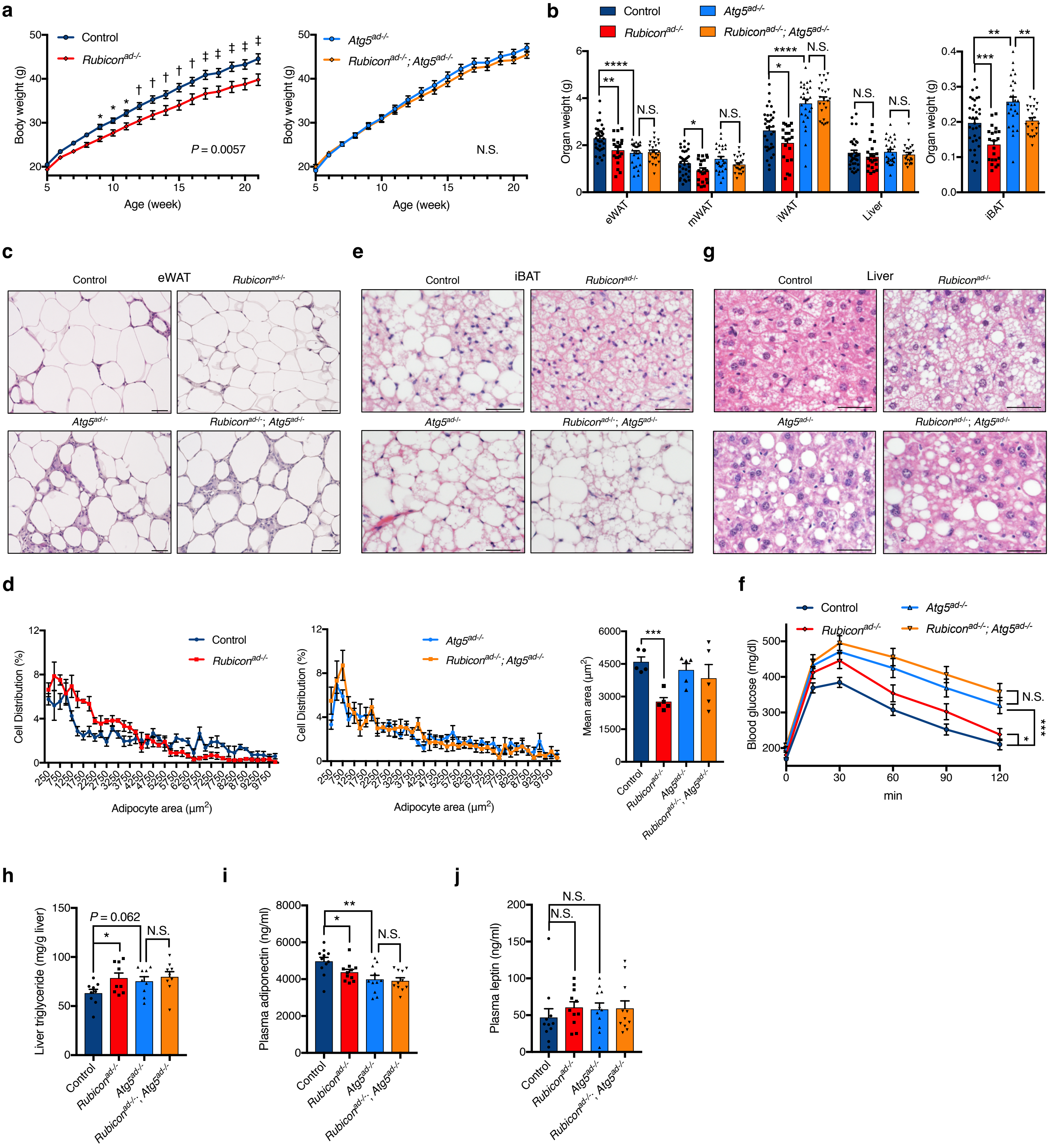

a
Control
Rubicon^ad-/-
Body weight (g)
Age (week)
P = 0.0057
Atg5^ad-/-
Rubicon^ad-/-; Atg5^ad-/-
N.S.
b
Control
Rubicon^ad-/-
Atg5^ad-/-
Rubicon^ad-/-; Atg5^ad-/-
Organ weight (g)
eWAT
mWAT
iWAT
Liver
iBAT
c
eWAT
Control
Rubicon^ad-/-
Atg5^ad-/-
Rubicon^ad-/-; Atg5^ad-/-
e
iBAT
g
Liver
d
Cell Distribution (%)
Adipocyte area (μm²)
Mean area (μm²)
f
Blood glucose (mg/dl)
min
h
Liver triglyceride (mg/g liver)
P = 0.062
i
Plasma adiponectin (ng/ml)
j
Plasma leptin (ng/ml)

**Supplementary Figure 4** Loss of *Rubicon* in adipocytes promotes HFD-induced glucose intolerance and hepatic steatosis. **a** Body weight chart for mice of the indicated genotypes on an HFD. IPGTT (Fig. 2c) and IPITT were performed at 17 and 19 weeks, respectively. Control, n = 34; *Rubicon*$^{ad-/-}$, n = 21; *Atg5*$^{ad-/-}$, n = 27; *Rubicon*$^{ad-/-}$; *Atg5*$^{ad-/-}$, n = 23. **b** Organ weight of the eWAT, mWAT, iWAT, iBAT, and liver from 21-week-old mice of the indicated genotypes, described in **a**. Control, n = 34; *Rubicon*$^{ad-/-}$, n = 21; *Atg5*$^{ad-/-}$, n = 27; *Rubicon*$^{ad-/-}$;*Atg5*$^{ad-/-}$, n = 23. **c, e, g** Representative images of H&E staining of eWAT (**c**), iBAT (**e**), and liver (**g**) sections from mice of the indicated genotypes on an HFD. Scale bars, 50 μm. n = 5 mice with similar results. **d** Distribution of adipocyte area in eWAT sections in **c**. n = 5 mice. Quantification of mean adipocyte area is shown in the graphs at right. **f** Glucose tolerance test in mice of the indicated genotypes on an HFD. Whole-body glucose levels were measured at 15, 30, 60, 90, and 120 min. Control, n = 34; *Rubicon*$^{ad-/-}$, n = 21; *Atg5*$^{ad-/-}$, n = 27; *Rubicon*$^{ad-/-}$;*Atg5*$^{ad-/-}$, n = 23. **h** Liver triglyceride levels in 21-week-old mice of the indicated genotypes on an HFD. n = 9 mice. **i, j** Plasma adiponectin (**i**) and leptin (**j**) levels in 21-week-old mice of the indicated genotypes on an HFD. n = 11 mice. Error bars indicate means ± SEM. Data were analysed by two-tailed Student's *t*-test (**b, d, h–j**) or two-way repeated-measures ANOVA followed by Fisher's LSD test (**a, f**). *P* value from top to bottom and left to right: 0.0057, 0.5419 (**a**), < 0.0001, 0.0028, 0.7891, 0.0348, 0.0717, < 0.0001, 0.0270, 0.5975, 0.3599, 0.4011, 0.0033, 0.0006, 0.0023 (**b**), 0.0003 (**d**), 0.3370, 0.0001, 0.0492 (**f**), 0.0622, 0.0313, 0.5392 (**h**), 0.0052, 0.0392, 0.7791 (**i**), 0.4651, 0.3445 (**j**). **P* < 0.05, ** or †*P* < 0.01, *** or ‡*P* < 0.001, *****P* < 0.0001. N.S., not significant.

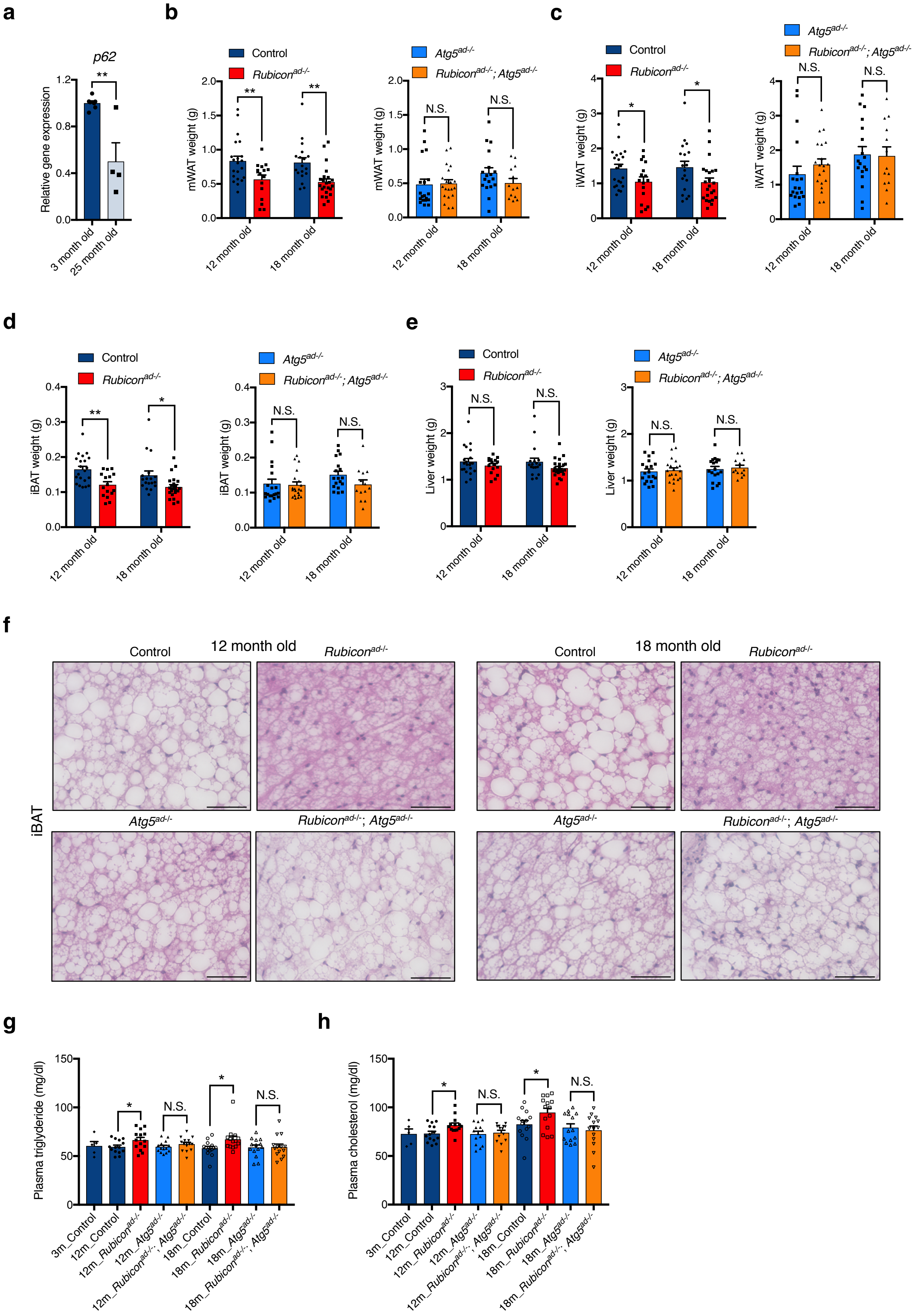

a
b
c
p62
**
Relative gene expression
1.2
0.8
0.4
0.0
3 month old
25 month old
Control
Rubicon$^{ad-/-}$
Atg5$^{ad-/-}$
Rubicon$^{ad-/-}$; Atg5$^{ad-/-}$
mWAT weight (g)
2.0
1.5
1.0
0.5
0.0
N.S.
*
iWAT weight (g)
4
3
2
1
0
12 month old
18 month old
d
e
iBAT weight (g)
0.4
0.3
0.2
0.1
0.0
Liver weight (g)
f
12 month old
18 month old
iBAT
g
h
Plasma triglyceride (mg/dl)
Plasma cholesterol (mg/dl)
150
100
50
0
3m_Control
12m_Control
12m_Rubicon$^{ad-/-}$
12m_Atg5$^{ad-/-}$
12m_Rubicon$^{ad-/-}$; Atg5$^{ad-/-}$
18m_Control
18m_Rubicon$^{ad-/-}$
18m_Atg5$^{ad-/-}$
18m_Rubicon$^{ad-/-}$; Atg5$^{ad-/-}$

**Supplementary Figure 5** Loss of *Rubicon* in adipocytes decreases fat mass in aged mice. **a** Relative mRNA expression of *p62* in eWAT depots of 3- and 25-month-old wild-type mice on an NCD. 3-month, n = 7; 25-month, n = 4. **b–e** mWAT weight (**b**), iWAT weight (**c**), iBAT weight (**d**), and liver weight (**e**) of mice of the indicated ages and genotypes on an NCD. 12-month Control, n = 21; 12-month *Rubicon*$^{ad-/-}$, n = 16; 12-month *Atg5*$^{ad-/-}$, n = 19; 12-month *Rubicon*$^{ad-/-}$; *Atg5*$^{ad-/-}$, n = 20. 18-month Control, n = 18; 18-month *Rubicon*$^{ad-/-}$, n = 23; 18-month *Atg5*$^{ad-/-}$, n = 18; 18-month *Rubicon*$^{ad-/-}$; *Atg5*$^{ad-/-}$, n = 13. **f** Representative images of H&E staining of iBAT sections from mice of the indicated ages and genotypes on an NCD. Scale bars, 50 µm. n = 5 mice with similar results. **g, h** Plasma triglyceride (**g**) and cholesterol (**h**) levels in mice of the indicated ages and genotypes on an NCD. n = 14 mice except for 3-month-old mice, for which n = 5. Error bars indicate means ± SEM. Data were analysed by two-tailed Student's *t*-test (**a–e, g, h**). *P* value from left to right: 0.0023 (**a**), 0.0093, 0.0011, 0.8736, 0.1687 (**b**), 0.0476, 0.0418, 0.3229, 0.9013 (**c**), 0.0015, 0.0157, 0.8198, 0.0996 (**d**), 0.2991, 0.1030, 0.7336, 0.6887 (**e**), 0.0392, 0.2743, 0.0258, 0.9185 (**g**), 0.0161, 0.7036, 0.0480, 0.6406 (**h**). $^*P < 0.05$, $^{**}P < 0.01$. N.S., not significant.

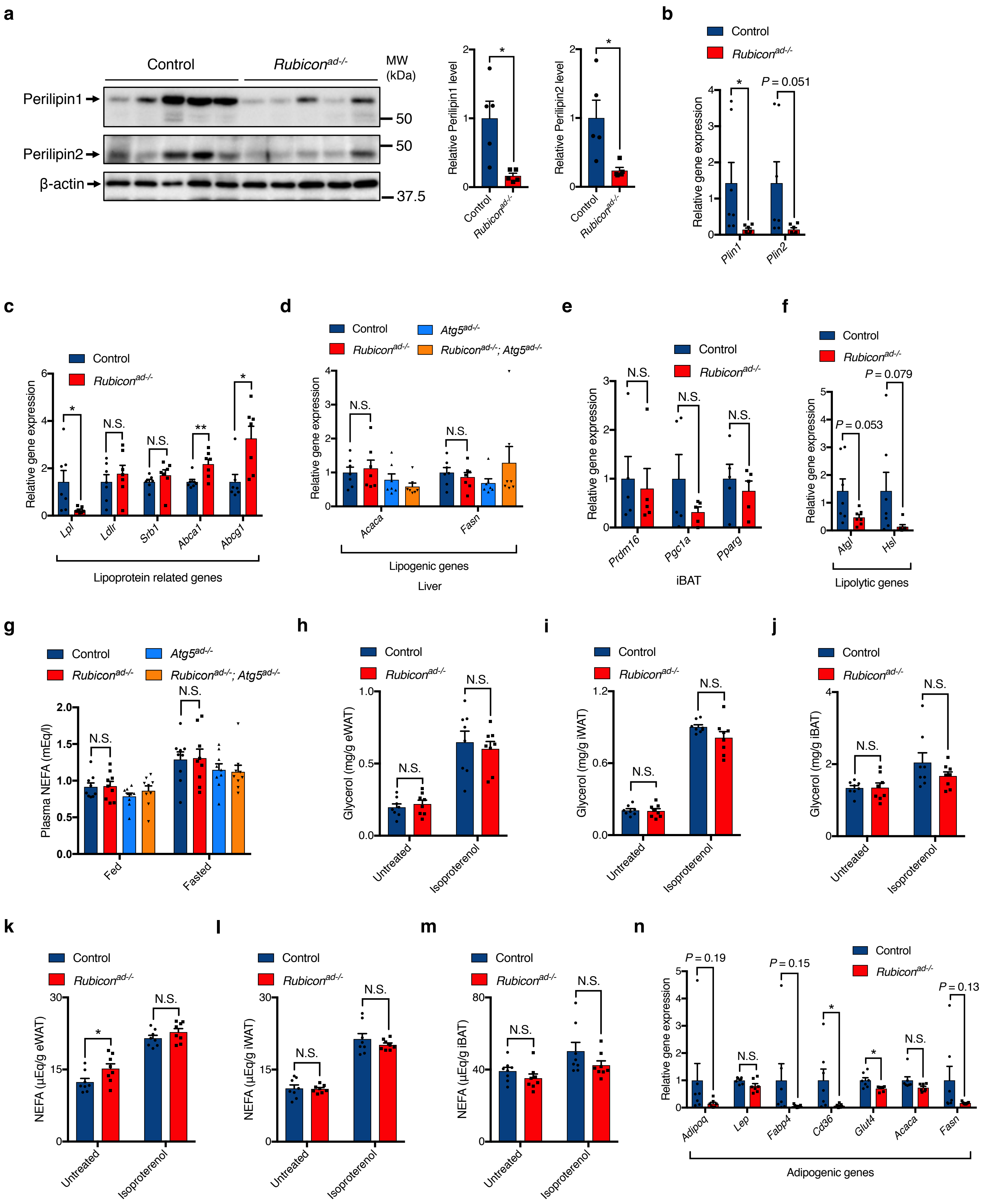
a
Control
Rubicon$^{ad-/-}$
MW (kDa)
Perilipin1
Perilipin2
β-actin
50
50
37.5
Relative Perilipin1 level
Relative Perilipin2 level
Control
Rubicon$^{ad-/-}$
*
b
Control
Rubicon$^{ad-/-}$
Relative gene expression
P = 0.051
Plin1
Plin2
c
Control
Rubicon$^{ad-/-}$
Relative gene expression
N.S.
**
Lpl
Ldlr
Srb1
Abca1
Abcg1
Lipoprotein related genes
d
Control
Rubicon$^{ad-/-}$
Atg5$^{ad-/-}$
Rubicon$^{ad-/-}$; Atg5$^{ad-/-}$
Relative gene expression
Acaca
Fasn
Lipogenic genes
Liver
e
Control
Rubicon$^{ad-/-}$
Relative gene expression
Prdm16
Pgc1a
Pparg
iBAT
f
Control
Rubicon$^{ad-/-}$
Relative gene expression
P = 0.053
P = 0.079
Atgl
Hsl
Lipolytic genes
g
Control
Rubicon$^{ad-/-}$
Atg5$^{ad-/-}$
Rubicon$^{ad-/-}$; Atg5$^{ad-/-}$
Plasma NEFA (mEq/l)
Fed
Fasted
h
Glycerol (mg/g eWAT)
Untreated
Isoproterenol
i
Glycerol (mg/g iWAT)
j
Glycerol (mg/g iBAT)
k
NEFA (µEq/g eWAT)
l
NEFA (µEq/g iWAT)
m
NEFA (µEq/g iBAT)
n
Control
Rubicon$^{ad-/-}$
Relative gene expression
P = 0.19
P = 0.15
P = 0.13
Adipoq
Lep
Fabp4
Cd36
Glut4
Acaca
Fasn
Adipogenic genes

**Supplementary Figure 6** Analysis of lipid metabolism pathways in adipose-specific *Rubicon*-knockout mice. **a** Immunoblotting to detect Perilipin1 and Perilipin2 in eWAT depots of 21-week-old mice of the indicated genotypes on an NCD. n = 5 mice. Quantification of Perilipin1 and Perilipin2 levels is shown in the graphs at right. **b, c, f** Relative mRNA expression of Perilipin genes (**b**), lipoprotein-related genes (**c**), and lipolytic genes (**f**) in eWAT depots of 21-week-old mice of the indicated genotypes on an NCD. n = 7 mice. **d** Relative mRNA expression of lipogenic genes in the liver of 21-week-old mice of the indicated genotypes on an NCD. n = 7 mice. **e** Relative mRNA expression of *Prdm16*, *Pgc1a*, and *Pparg* in iBAT depots of 21-week-old mice of the indicated genotypes on an NCD. n = 5 mice. **g** Plasma NEFA levels in fed or 24-hour–fasted 5–6-month-old mice of the indicated genotypes on an NCD. n = 9 mice. **h–j** *Ex vivo* lipolysis assay based on glycerol release in eWAT (**h**), iWAT (**i**), and iBAT (**j**) depots of 5-month-old mice of the indicated genotypes on an NCD. n = 8 biologically independent samples. **k–m** *Ex vivo* lipolysis assay based on NEFA release in eWAT (**k**), iWAT (**l**), and iBAT (**m**) depots of 5-month-old mice of the indicated genotypes on an NCD. n = 8 biologically independent samples. **n** Relative mRNA expression of adipogenic genes in eWAT depots of 3-month-old mice of the indicated genotypes on an NCD. n = 7 mice. Error bars indicate means ± SEM. Data were analysed by two-tailed Student's *t*-test (**a–n**). *P* value from left to right: 0.0104, 0.0200 (**a**), 0.0436, 0.0515 (**b**), 0.0279, 0.4771, 0.2901, 0.0073, 0.0102 (**c**), 0.6838, 0.5088 (**d**), 0.7514, 0.2161, 0.5063 (**e**), 0.0527, 0.0787 (**f**), 0.8930, 0.9072 (**g**), 0.5339, 0.6229 (**h**), 0.8255, 0.1136 (**i**), 0.9563, 0.2267 (**j**), 0.0449, 0.2007 (**k**), 0.8827, 0.3314 (**l**), 0.2234, 0.1738 (**m**), 0.1931, 0.0580, 0.1485, 0.0454, 0.0148, 0.0824, 0.1321 (**n**). $*P < 0.05$, $**P < 0.01$. N.S., not significant.

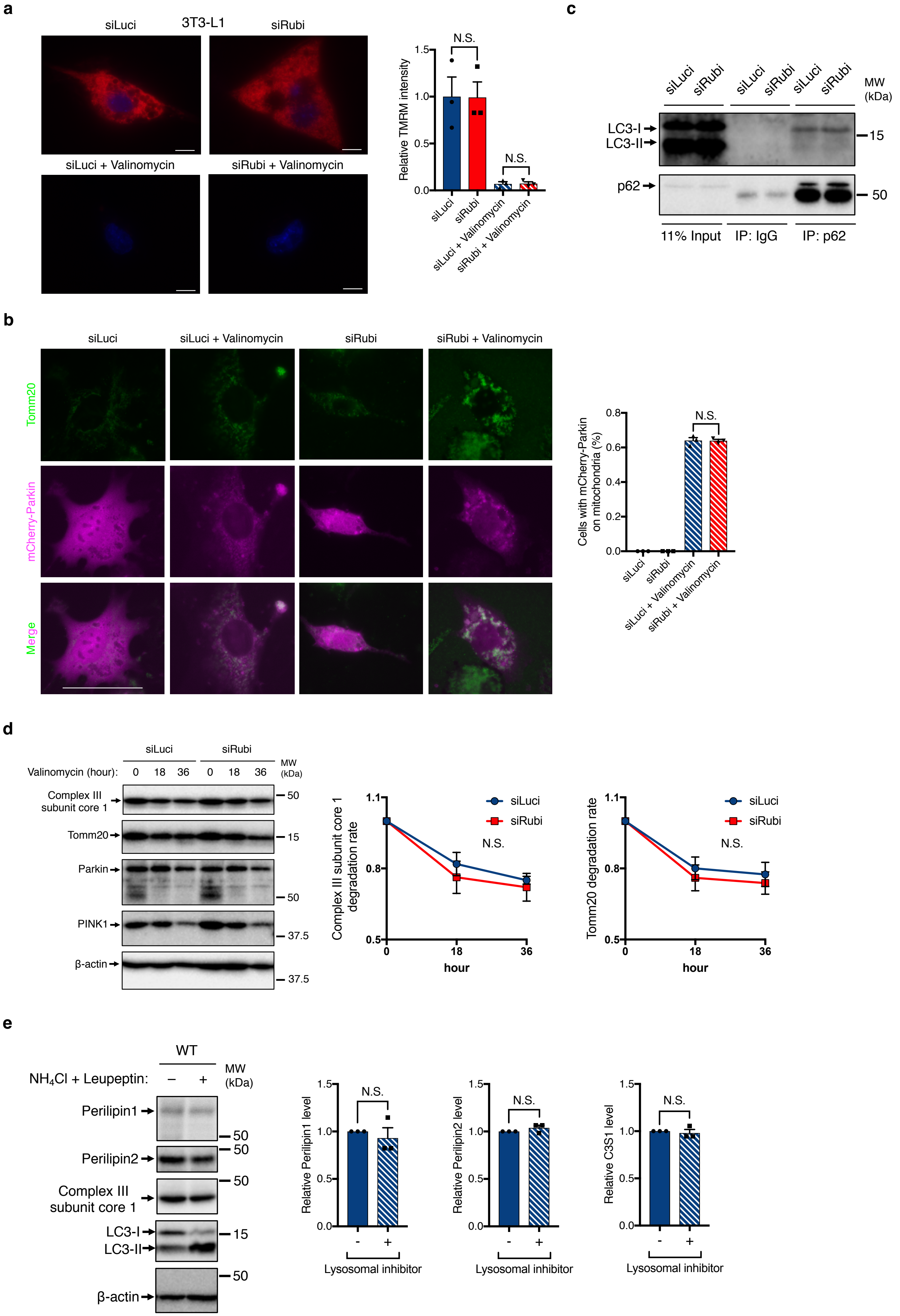

a
3T3-L1
siLuci
siRubi
siLuci + Valinomycin
siRubi + Valinomycin
Relative TMRM intensity
N.S.
b
siLuci
siLuci + Valinomycin
siRubi
siRubi + Valinomycin
Tomm20
mCherry-Parkin
Merge
Cells with mCherry-Parkin on mitochondria (%)
c
siLuci
siRubi
MW (kDa)
LC3-I
LC3-II
p62
11% Input
IP: IgG
IP: p62
d
Valinomycin (hour): 0 18 36 0 18 36
Complex III subunit core 1
Tomm20
Parkin
PINK1
β-actin
Complex III subunit core 1 degradation rate
Tomm20 degradation rate
hour
e
WT
NH4Cl + Leupeptin: – +
Perilipin1
Perilipin2
Complex III subunit core 1
LC3-I
LC3-II
β-actin
Relative Perilipin1 level
Relative Perilipin2 level
Relative C3S1 level
Lysosomal inhibitor

**Supplementary Figure 7** Analysis of mitophagy in *Rubicon*-knockdown adipocytes. **a** Representative images of staining of TMRM and Hoechst33342 in the live *Luciferase*- or *Rubicon*-knockdown 3T3-L1 cells treated with or without 10 μM valinomycin for 3 hours. The live cells were stained with 20 nM TMRM for 30 min before valinomycin treatment. Hoechst33342 staining was carried out immediately before the observation. Knockdown was performed for 48 hours starting on day 8 after induction of differentiation. Scale bars, 10 μm. n = 3 independent experiments. Quantification of relative TMRM intensity is shown in the graphs at right. **b** Representative images of immunocytochemistry to detect Tomm20 in *Luciferase*- or *Rubicon*-knockdown 3T3-L1 cells treated with or without 10 μM valinomycin for 3 hours. Knockdown was performed for 48 hours starting on day 8 after induction of differentiation. pmCherry-Parkin was transfected for 24 hours starting on day 9. Cells were harvested at day 10. Scale bars, 50 μm. n = 3 independent experiments. Quantification of mCherry-Parkin on mitochondria is shown in the graphs at right. **c** Immunoprecipitation assay. *Luciferase*- or *Rubicon*-knockdown 3T3-L1 cells were lysed and immunoprecipitated with control IgG or anti-p62 antibody. Knockdown was performed for 48 hours starting on day 8 following induction of differentiation. Precipitates were subjected to immunoblotting with the indicated antibodies. **d** Immunoblotting to detect the indicated proteins in *Luciferase*- or *Rubicon*-knockdown 3T3-L1 cells treated with 10μM valinomycin for the indicated time points. Knockdown was performed for 48 hours starting on day 8 following induction of differentiation. n = 3 independent experiments. **e** Immunoblotting to detect the indicated proteins in wild-type eWAT depots explanted in DMEM, treated with or without 20 mM ammonium chloride and 200 μM leupeptin for 2 hours. n = 3 independent experiments. Quantification data is shown in the graphs at the right of each blot. Error bars indicate means ± SEM. Data were analysed by two-tailed Student's *t*-test (**a, b, e**) or two-

way ANOVA followed by Fisher's LSD test (**d**). *P* value from left to right: 0.9781, 0.9311 (**a**), 0.9400 (**b**), 0.3909, 0.6480 (**d**), 0.5584, 0.2354, 0.6350 (**e**). N.S., not significant.

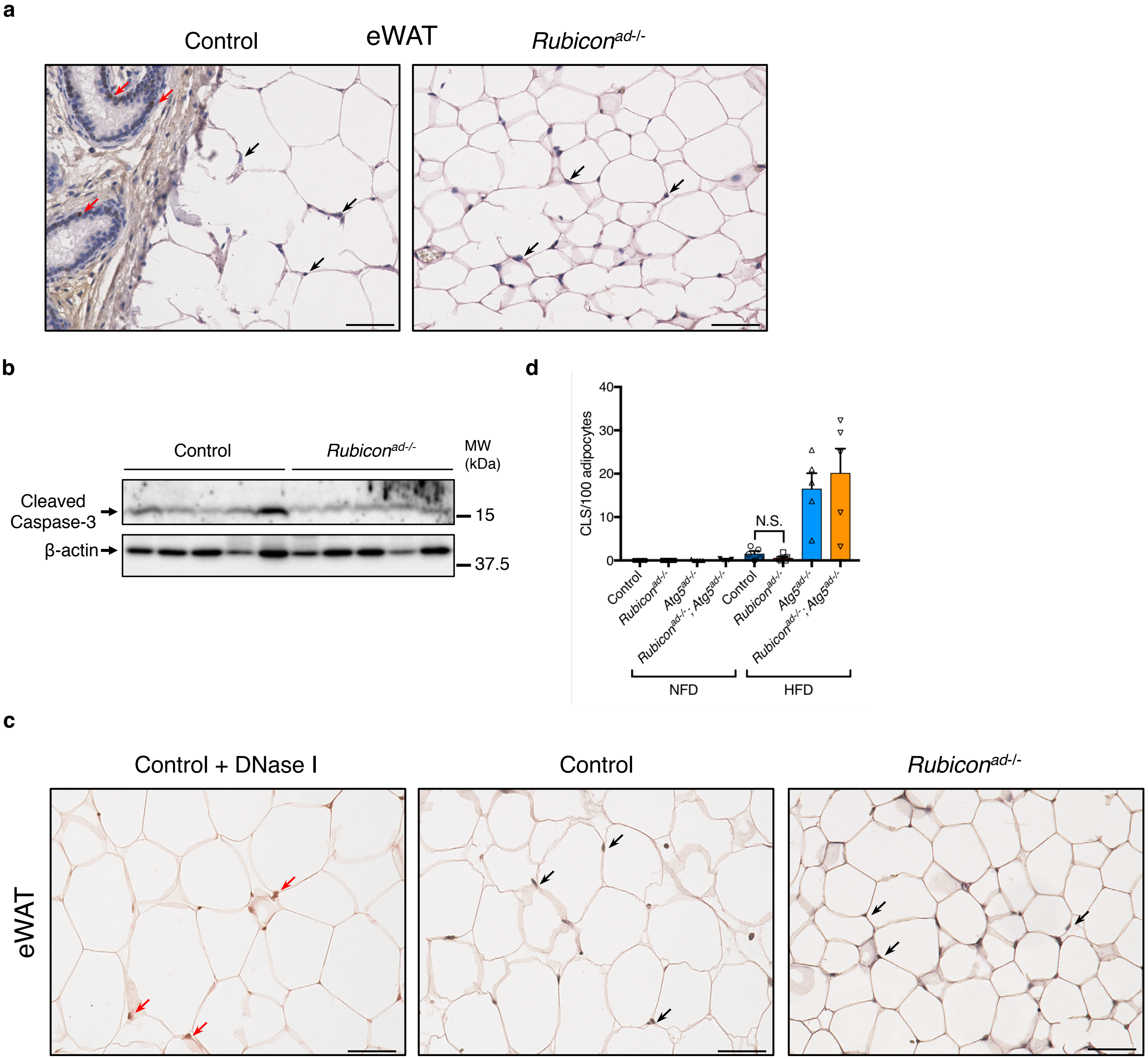

a
Control
eWAT
Rubicon^ad-/-
b
Control
Rubicon^ad-/-
MW (kDa)
Cleaved Caspase-3
β-actin
15
37.5
d
CLS/100 adipocytes
40
30
20
10
0
N.S.
Control
Rubicon^ad-/-
Atg5^ad-/-
Rubicon^ad-/-; Atg5^ad-/-
NFD
HFD
c
Control + DNase I
Control
Rubicon^ad-/-
eWAT

**Supplementary Figure 8** Analysis of cell death in adipose-specific *Rubicon*-knockout mice. **a** Representative images of immunohistochemistry to detect PCNA in eWAT sections from mice of the indicated genotypes on an NCD. Red and black arrows indicate positive and negative staining, respectively. Scale bars, 50 μm. n = 5 mice with similar results. **b** Immunoblotting to detect Cleaved Caspase-3 in eWAT depots of 21-week-old mice of the indicated genotypes on an NCD. n = 5 mice. **c** Representative images of TUNEL staining of eWAT sections from mice of the indicated genotypes on an NCD. Red and black arrows indicate positive and negative staining, respectively. Scale bars, 50 μm. DNase I–treated eWAT sections from control mice were used as a positive control. n = 5 mice with similar results. **d** Quantification of CLS in eWAT sections from mice of the indicated genotypes on an NCD or HFD. n = 5 mice. Error bars indicate means ± SEM. Data were analysed by two-tailed Student's *t*-test (**d**). *P* value: 0.2137 (**d**). N.S., not significant.

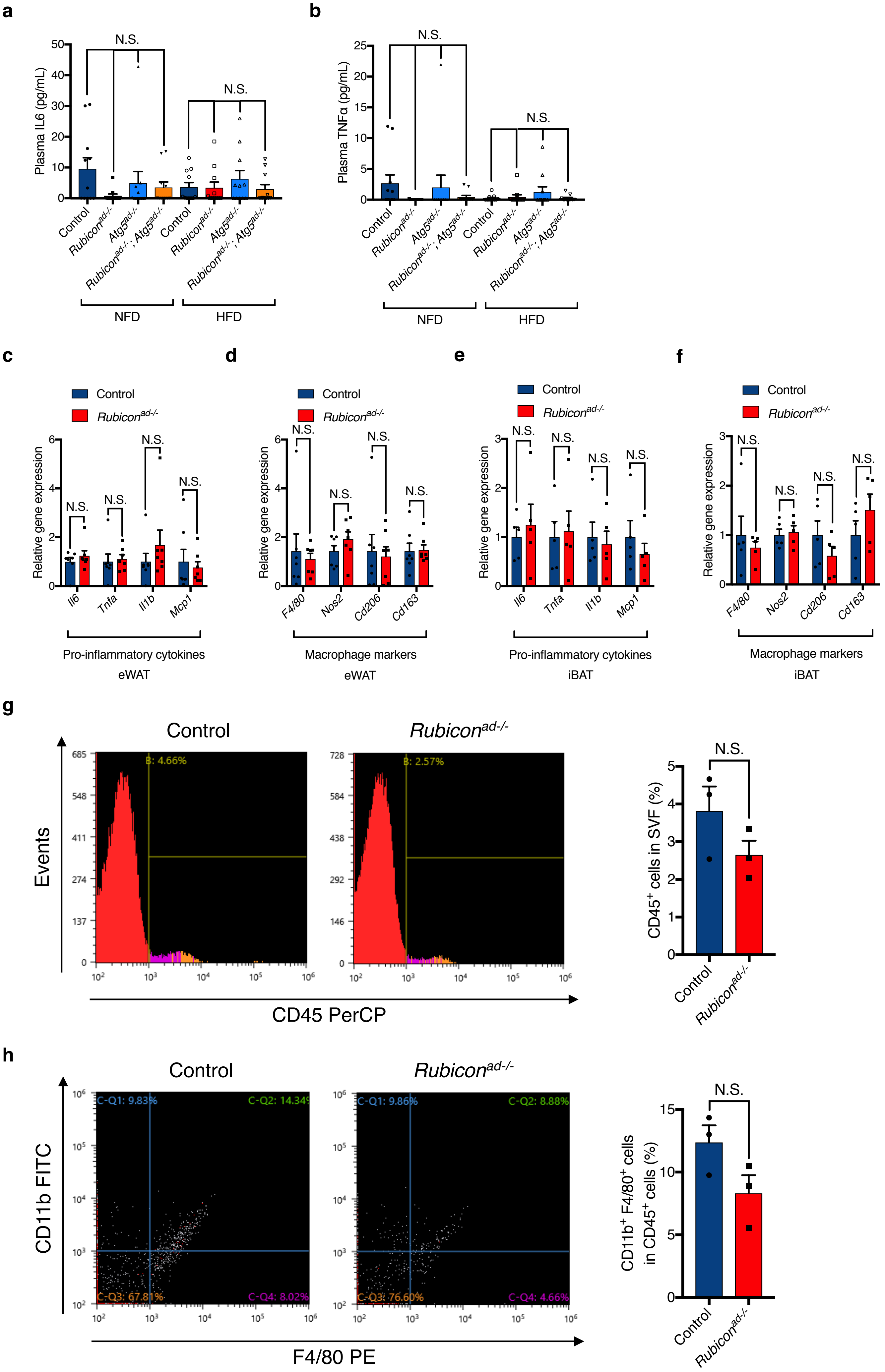
a
b
Plasma IL6 (pg/mL)
Plasma TNFα (pg/mL)
N.S.
Control
Rubiconad-/-
Atg5ad-/-
Rubiconad-/-; Atg5ad-/-
NFD
HFD
c
d
e
f
Control
Rubiconad-/-
Relative gene expression
Il6
Tnfa
Il1b
Mcp1
F4/80
Nos2
Cd206
Cd163
Pro-inflammatory cytokines
eWAT
Macrophage markers
iBAT
g
Events
B: 4.66%
B: 2.57%
CD45 PerCP
CD45+ cells in SVF (%)
h
CD11b FITC
C-Q1: 9.83%
C-Q2: 14.34%
C-Q3: 67.81%
C-Q4: 8.02%
C-Q1: 9.86%
C-Q2: 8.88%
C-Q3: 76.60%
C-Q4: 4.66%
F4/80 PE
CD11b+ F4/80+ cells in CD45+ cells (%)

**Supplementary Figure 9** Analysis of inflammation in adipose-specific *Rubicon*-knockout mice. **a, b** Plasma IL6 (**a**) and TNFα (**b**) levels in 21-week-old mice of the indicated genotypes on an NCD or HFD. n = 11 mice. **c–f** Relative mRNA expression of pro-inflammatory cytokines (**c, e**) or macrophage markers (**d, f**) in eWAT (**c, d**) or iBAT (**e, f**) depots of 21-week-old mice of the indicated genotypes on an NCD. n = 7 mice (**c, d**). n = 5 mice (**e, f**). **g** Representative flow cytometry histograms of the SVF cells stained with CD45 PerCP. The SVF cells were separated from the eWAT depots of 5–6-month-old mice of the indicated genotypes on an NCD. n = 3 biologically independent samples. Quantification of the rate of $CD45^+$ cells in SVF is shown in the graphs at right. **h** Representative flow cytometry dot plots of the $CD45^+$ cells stained with CD11b FITC and F4/80 PE. The $CD45^+$ cells were described in **h**. n = 3 biologically independent samples. Quantification of the rate of $CD11b^+$ $F4/80^+$ cells in $CD45^+$ cells is shown in the graphs at right. Error bars indicate means ± SEM. Data were analysed by two-tailed Student's *t*-test (**c–h**) or one-way ANOVA followed by Tukey's test (**a, b**). *P* value from left to right: 0.1740, 0.6054 (**a**), 0.3760, 0.3481 (**b**), 0.3869, 0.7529, 0.3441, 0.6753 (**c**), 0.6791, 0.2279, 0.7885, 0.8864 (**d**), 0.6057, 0.8222, 0.7227, 0.4082 (**e**), 0.5418, 0.7434, 0.2564, 0.2690 (**f**), 0.1956 (**g**), 0.1107 (**h**). N.S., not significant.

**Supplementary Table 1** Sequences of qRT-PCR primers.

| **Gene** | **Direction** | **Sequence** |
|---|---|---|
| *Rubicon* | Forward | CTCATCCATGACCAGGTGTG |
| *Rubicon* | Reverse | GTCGCTCTCATGCAAACTGA |
| *p62* | Forward | GCCAGAGGAACAGATGGAGT |
| *p62* | Reverse | TCCGATTCTGGCATCTGTAG |
| *Adipoq* | Forward | GATGGCAGAGATGGCACTCC |
| *Adipoq* | Reverse | CTTGCCAGTGCTGCCGTCAT |
| *Lep* | Forward | GATGGACCAGACTCTGGCAG |
| *Lep* | Reverse | AGAGTGAGGCTTCCAGGACG |
| *Fabp4* | Forward | CCGCAGACGACAGGA |
| *Fabp4* | Reverse | CTCATGCCCTTTCATAAACT |
| *Cd36* | Forward | GATGTGGAACCCATAACTGGATTCAC |
| *Cd36* | Reverse | GGTCCCAGTCTCATTTAGCCACAGTA |
| *Glut4* | Forward | GCGGATGCTATGGGTCCTTA |
| *Glut4* | Reverse | GTCCGGCCTCTGGTTTCAG |
| *Acaca* | Forward | GAGGTACCGAAGTGGCATCC |
| *Acaca* | Reverse | GTGACCTGAGCGTGGGAGAA |
| *Fasn* | Forward | GAGAAGCCATGTGGGGAAGATTTC |
| *Fasn* | Reverse | TGAGCAGGGACAGGACAAGAC |
| *Il6* | Forward | ACAACCACGGCCTTCCCTACTT |
| *Il6* | Reverse | CACGATTTCCCAGAGAACATGTG |

| | | |
|---|---|---|
| *Tnfa* | Forward | TGTGCTCAGAGCTTTCAACAAC |
| *Tnfa* | Reverse | GCCCATTTGAGTCCTTGATG |
| *Il1b* | Forward | TCGCTCAGGGTCACAAGAAA |
| *Il1b* | Reverse | CCATCAGAGGCAAGGAGGAA |
| *Mcp1* | Forward | CCACTCACCTGCTGCTACTCAT |
| *Mcp1* | Reverse | TGGTGATCCTCTTGTAGCTCTCC |
| *Prdm16* | Forward | AAACGGCCTGAGATCCAAGAC |
| *Prdm16* | Reverse | TCCTCTACGTCCTCTGGCTTTG |
| *Pgc1a* | Forward | CAGCCTCTTTGCCCAGATCT |
| *Pgc1a* | Reverse | CCGCTAGCAAGTTTGCCTCA |
| *Pparg* | Forward | ATCTTAACTGCCGGATCCACAA |
| *Pparg* | Reverse | GCCCAAACCTGATGGCATT |
| *F4/80* | Forward | CTGCACCTGTAAACGAGGCTT |
| *F4/80* | Reverse | GCAGACTGAGTTAGGACCACAA |
| *Nos2* | Forward | CCAAGCCCTCACCTACTTCC |
| *Nos2* | Reverse | CTCTGAGGGCTGACACAAGG |
| *Cd206* | Forward | GTTCACCTGGAGTGATGGTTCTC |
| *Cd206* | Reverse | AGGACATGCCAGGGTCACCTTT |
| *Cd163* | Forward | GGCTAGACGAAGTCATCTGCAC |
| *Cd163* | Reverse | CTTCGTTGGTCAGCCTCAGAGA |
| *Ldlr* | Forward | GGGCCTCTGTCTGGTGTTTA |
| *Ldlr* | Reverse | AGCAGGCTGGATGTCTCTGT |
| *Srb1* | Forward | GGGCTCGATATTGATGGAGA |
| *Srb1* | Reverse | GGAAGCATGTCTGGGAGGTA |

| | | |
|---|---|---|
| *Abca1* | Forward | AGGCCGCACCATTATTTGTC |
| *Abca1* | Reverse | GGCAATTCTGTCCCCAAGGAT |
| *Abcg1* | Forward | ATTTCATCGTCCTGGGCATCT |
| *Abcg1* | Reverse | CGGATTTTGTATCTGAGGACGAA |
| *Lpl* | Forward | TTTATCCCAATGGAGGCACTTT |
| *Lpl* | Reverse | CAATCACACGGATGGCTTCTC |
| *Plin1* | Forward | GGGACCTGTGAGTGCTTCC |
| *Plin1* | Reverse | GTATTGAAGAGCCGGGATCTTTT |
| *Plin2* | Forward | GTGGAAAGGACCAAGTCTGTG |
| *Plin2* | Reverse | GACTCCAGCCGTTCATAGTTG |
| *36b4* | Forward | GCTCCAAGCAGATGCAGCA |
| *36b4* | Reverse | CCGGATGTGAGGCAGCAG |
